# Safe Treatment of Trigger Thumb With Longitudinal Anatomic Landmarks

**Published:** 2010-10-07

**Authors:** Ron Hazani, Josh Elston, Ryan D. Whitney, Jeremiah Redstone, Saeed Chowdhry, Bradon J. Wilhelmi

**Affiliations:** Division of Plastic and Reconstructive Surgery, University of Louisville School of Medicine, Kentucky

## Abstract

**Objective:** Stenosing tenosynovitis of the thumb flexor tendon sheath is also known as trigger thumb. It is an inflammatory process that involves the flexor tendon sheath at the A1 pulley. Successful percutaneous or open treatment of trigger thumb depends on the ability of the clinician to properly predict the location of the A1 pulley. Longitudinal anatomic landmarks can facilitate safe treatment for the trigger thumb while circumventing injury to the neurovascular bundles. **Methods:** Fourteen fresh cadaveric hands were dissected to identify surface landmarks corresponding to the oblique course of the flexor pollicis longus tendon at the level of the A1 pulley. **Results:** The longitudinal landmarks for the A1 pulley of the thumb are the palpable hook of the hamate and the midline of the thumb interphalangeal (IP) crease. Other bony prominences, such as the pisiform bone did not serve as effective landmarks while the thumb was in an abducted position. **Conclusion:** we encourage the use longitudinal anatomic landmarks to predict the location of the thumb A1 pulley. The hook of the hamate and the midline at the palmar interphalangeal crease are reliable landmarks for safe release of the A1 pulley while avoiding inadvertent injury to adjacent structures.

Trigger thumb is noninfectious inflammation of the flexor tendon sheath of the thumb. The flexor tendon travels in a fibroosseous tunnel between the metacarpal and interphalangeal bones. Irritation or inflammation of this tunnel can occur as a result of repetitive use, thus preventing smooth gliding of the tendon under the A1 pulley. This point of constriction can cause the thumb to lock in a flexed position.^1^ Nonoperative modalities include splinting, steroid injection, local anesthetic injection, and behavior modification.^2^^-^9 However, overall results with nonoperative management have been variable and disappointing.^1^ Operative treatment of trigger thumb includes incision of the A1 pulley by percutaneous or open technique. Success rates have proven to be higher with surgical treatment, but so are complication rates.^10^

Anatomic landmarks for the A1 pulley of the thumb can facilitate complete transection of the pulley while circumventing injury to the critical oblique pulley and the neurovascular bundles. The purpose of this study is to identify longitudinal landmarks to pinpoint the exact location the thumb A1 pulley and avoid damage to the adjacent neurovascular bundles. Our proposed landmarks are the thumb midline at the interphalangeal crease, the palpable hook of the hamate, and the pisiform bone (Fig 1). Dissection of the A1 pulley in reference to our anatomic landmarks would identify the vector along which the A1 pulley is located.

## METHODS

**Figure 2:**
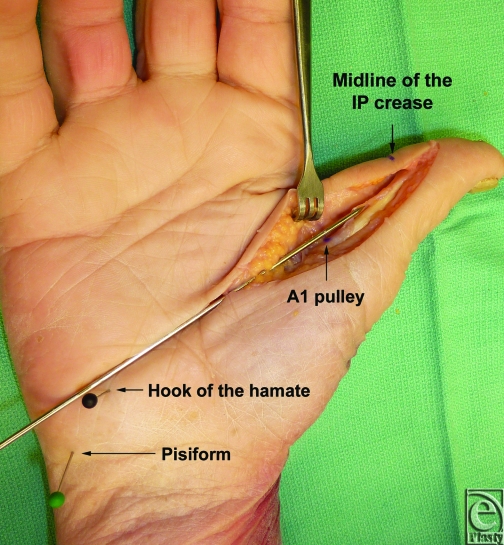
A longitudinal line from the hook of the hamate to the midline of the thumb interphalangeal (IP) crease marks the accurate location of the A1 pulley along the course of the flexor pollicis longus tendon.

Fourteen fresh cadaveric hands were dissected with the aid of loupe magnification. The thumb was placed in a fully abducted position. Skin flaps were developed in a longitudinal fashion, exposing the flexor tendon sheath of the thumb from the flexor pollicis longus tendon insertion to the A1 pulley. At the level of the pulley, a longitudinal line was then identified crossing from the midline of the interphalangeal crease to the hand palmar surface. We then identified a bony anatomic landmark at the palm that corresponds with our longitudinal line (Fig 2).

## RESULTS

In all cadaveric specimens, a longitudinal line from the midline of the thumb interphalangeal crease to the hook of the hamate corresponded to the flexor pollicis longus tendon at the A1 pulley. The line appears to be passing through the midpoint of the hook of the hamate. Other bony prominences, such as the pisiform bone, did not serve as potential landmarks, while the thumb was in an abducted position. Transection of the A1 pulley along the course of our longitudinal line did not injure the neurovascular bundles.

## DISCUSSION

Trigger finger is an inflammatory condition that involves the flexor tendon sheath of the digits. It can involve any digit but typically affects the thumb and ring finger of the dominant hand. A tendon nodule develops at the site of constriction into the fibrous flexor sheath. It is this nodule that catches the proximal end of the fibrous sheath at the A1 pulley with finger flexion, causing the symptoms of trigger finger with extension. Initially, patients experience intermittent pain, swelling, and triggering of the involved digit. In the most severe state, the digit becomes locked in the flexed position. Clinically, the tendon nodule and triggering is often palpable.^1^

Stenosing tenosynovitis can be managed with nonoperative or surgical modalities. Steroid injections have been described for the early treatment of trigger thumb. The procedure involves instilling a steroid with or without local anesthetic into the lumen of the tendon sheath just proximal to the A1 pulley; however, Kamhin et al^11^ found that the injection filled the lumen in only 49% of injections. In addition, reported success rates with steroid injection demonstrated significant variance. Quinnell^12^ reported only a cure rate of 38% with steroid injection, whereas Rhoades et al^13^ reported a cure rate of only 55% with a single injection; however, the rate improved to 82% with an additional injection. The success of this technique is dependent upon the time of presentation. Kamhin et al^11^ found that the success rate for injection of steroid decreased from 93% to 41% for patients with long-standing disease. Moreover, several authors have recommended release for patients who have had symptoms for greater than 4 months.^14^ In general, failure of nonoperative treatment results in the need for surgical release.

Open division of the A1 pulley is typically performed with local anesthetic and sometimes with a general anesthetic. Surgical release of the A1 pulley has been shown to result in higher cure rates but is associated with higher complication rates. Satisfaction rates for open A1 pulley release have been reported to be from 60% to 100%.^10^,^15^^-^17 The complication rate for open release, including digital nerve injury, infection, stiffness, weakness, scar tenderness, and bowstringing of the flexor tendons, ranges from 7% to 28%. The reported rate of infection and digital nerve damage is as high as 12%.^15^^-^17

Given the potential for such a high complication rate, we propose the use of anatomic landmarks for the A1 pulley to facilitate a more reliable management of this entity. This approach can produce a safer treatment of trigger thumb, whether it is percutaneous or open. This study can map the location of the thumb A1 pulley accurately in a 2-dimensional axis. It should be noted that our findings are relevant to patients with supple thumb joints where the thumb can be placed in an abducted position. It is likely that these landmarks might not be relevant to patients with stiffness secondary to basilar joint arthritis or other disabling conditions.

In conclusion, the midline at the interphalangeal crease of the thumb and the palpable hook of the hamate are reliable longitudinal landmarks for the safe release of the A1 pulley while avoiding inadvertent injury to adjacent neurovascular structures. We encourage the use of these landmarks in the open or percutaneous approach to the treatment of trigger thumb.

## Figures and Tables

**Figure 1:**
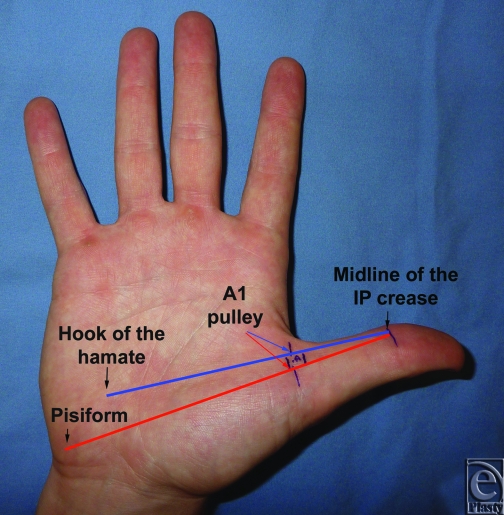
Proposed longitudinal anatomic landmarks for the thumb A1 pulley: the midline of the thumb interphalangeal (IP) crease, the hook of the hamate, and the pisiform. The red and blue longitudinal lines mark the possibility of locating of the A1 pulley along the course of the flexor pollicis longus tendon.

## References

[1] Wilhelmi BJ, Mowlavi A, Neumeister MW, Bueno R, Lee WP (2003). Safe treatment of trigger finger with longitudinal and transverse landmarks: an anatomic study of the border fingers for percutaneous release. Plast Reconstr Surg.

[2] Benson LS, Ptaszek AJ (1997). Injection versus surgery in the treatment of trigger finger. J Hand Surg Am.

[3] Brito JL, Rozental TD (2010). Corticosteroid injection for idiopathic trigger finger. J Hand Surg Am.

[4] Chambers RG (2009). Corticosteroid injections for trigger finger. Am Fam Physician.

[5] Colbourn J, Heath N, Manary S, Pacifico D (2008). Effectiveness of splinting for the treatment of trigger finger. J Hand Ther.

[6] Makkouk AH, Oetgen ME, Swigart CR, Dodds SD (2008). Trigger finger: etiology, evaluation, and treatment. Curr Rev Musculoskelet Med.

[7] Ryzewicz M, Wolf JM (2006). Trigger digits: principles, management, and complications. J Hand Surg Am.

[8] Swezey RL (1999). Trigger finger splinting. Orthopedics.

[9] Ring D, Lozano-Calderon S, Shin R, Bastian P, Mudgal C, Jupiter J (2008). A prospective randomized controlled trial of injection of dexamethasone versus triamcinolone for idiopathic trigger finger. J Hand Surg Am.

[10] Thorpe AP (1988). Results of surgery for trigger finger. J Hand Surg Br.

[11] Kamhin M, Engel J, Heim M (1983). The fate of injected trigger fingers. Hand.

[12] Quinnell RC (1980). Conservative management of trigger finger. Practitioner.

[13] Rhoades CE, Gelberman RH, Manjarris JF (1984). Stenosing tenosynovitis of the finger and thumb. Clin Orthop.

[14] Eastwood DM, Gupta KJ, Johnson DP (1992). Percutaneous release of the trigger finger: an office procedure. J Hand Surg Am.

[15] Bonnici AV, Spencer JD (1988). A survey of ‘trigger finger’ in adults. J Hand Surg Br.

[16] Turowski GA, Zdankiewicz PD, Thomson JG (1997). The results of surgical treatment of trigger finger. J Hand Surg Am.

[17] Hodgkinson JP, Unwin A, Noble J, Binns MS (1988). Retrospective study of 120 trigger digits treated surgically. J R Coll Surg Edinb.

